# Social exclusion and mental health of youths affected by parental HIV/AIDS in China: Based on a serial mediating model

**DOI:** 10.1371/journal.pone.0327089

**Published:** 2025-07-02

**Authors:** Jiaojiao Wan, Lili Ji, ZheQian Wang, Junfeng Zhao, Xiaoming Li

**Affiliations:** 1 School of Educational Science, Anhui Normal University, Wuhu, China; 2 College of Nursing and Health, Institute of Nursing and Health, Henan University, Kaifeng, China; 3 Publicity Department, Anhui Normal University, Wuhu, China; 4 School of Psychology, Institute of Behavior and Psychology, Henan University, Kaifeng, China; 5 Department of Health Promotion, Education, and Behavior, University of South Carolina, Columbia, South Carolina, United States of America; University of Haifa Faculty of Social Welfare and Health Sciences, ISRAEL

## Abstract

The mental health of youth living with patients with HIV needs focused attention. The primary source of psychological distress they experience may stem from social exclusion. Therefore, the current study was designed to explore the relationship between social exclusion and mental health among youths affected by parental HIV/AIDS in rural China.

**Methods:**

297 youths affected by parental HIV/AIDS were recruited to completed questionnaires of social exclusion, perceived stress, future orientation and mental health. The bootstrap method was used to examine the mediation effects.

**Results:**

It showed that: (1) the social exclusion (*M* = 32.48, *SD* = 15.25) significantly and negatively predicted the mental health (*M* = 93.27, *SD* = 19.08). (2) Perceived stress (*M* = 39.28, *SD* = 6.35) mediated the negative effect of the social exclusion and mental health. (3) Future orientation (*M* = 57.44, *SD* = 8.15) mediated the relationship between social exclusion and mental health. (4) Perceived stress and future orientation could play a chain-mediating role in the mechanism of social exclusion affecting the mental health.

**Conclusions:**

Results of this study support the Stress and Coping Theory (SCT) and demonstrate the damaging effect of perceived stress and the protective effect of future orientation in mediating the relationship between social exclusion and mental health among youths affected by parental HIV/AIDS in China. Future mental health promotion and intervention efforts targeting these youths or other youths with early childhood adversity should include components that could mitigate the negative impact of social exclusion on their lives.

## Introduction

Millions of people worldwide are affected by HIV/AIDS. By 2023, about 39.9 million people were living with HIV, and about 42.3 million people had died of AIDS-related illness, resulting in a large number of orphans worldwide [1]. In China, the epidemic remains a significant public health challenge, with over 1.22 million cumulative reported HIV/AIDS cases by 2022 [2]. Studies have found that young people with parents infected or died of HIV/AIDS (“youths affected by parental HIV/AIDS”) would have psychological problems such as depression, anxiety, anger, and post-traumatic stress symptoms, which could cause some social adaptation problems [3–5]. Most psychological issues manifested in older age were the results of adverse experiences in early childhood [6]. Youths affected by parental HIV/AIDS might elevate their risk for poor psychological outcomes and face additional challenges as they transitioned through childhood into young adulthood.

### Social exclusion affects the mental health of youths affected by parental HIV/AIDS

Socioecology theory emphasized social environment as one of the important factors affecting the psychological development of individuals [7]. Previous research consistently found that youths affected by parental HIV/AIDS, compared to their peers who did not experience HIV/AIDS-related illness and death in their families, might be more vulnerable to hostility from extended families and communities [8], being rejected by schools and workplaces [9], and even being discriminated against in sexual relationship or marriage [10]. It is no wonder that the lay public and scholars alike are keen to understand and explore the effects and mechanism of negative social environmental factors (e.g., social exclusion, HIV-related stigma, and peer victimization) on mental health among children or youths affected by parental HIV/AIDS [11,12]. Answering these questions is helpful to explore relevant intervention conditions in order to promote the psychosocial adaptation of these youths as well as other youths who experienced early childhood adversity.

Humans have a strong demand for stable social belonging [13]. Conceptualized as being excluded by individuals or social groups [14], social exclusion, as a negative social environment factor, thwarts this fundamental need and threatens individual positive development. On the one hand, social exclusion could impair emotional function. Williams [15] proposed that social exclusion will directly threaten the satisfaction of the basic needs of individuals, which will affect their emotional management and increase the risk of depression [15,16]. On the other hand, the emotional distress caused by social exclusion could further produce a short-term impairment of cognitive functioning and then endanger mental health. Baumeister et al. [17] designed three experiments to explore the negative cognitive effect of social exclusion, and they found that people exhibited significant cognitive decrements after they were told that they were likely to end up being lonely in life. These cognitive impairments might affect the individual mental health and social adaptation [18]. As a result, this research proposes Hypothesis 1:

*H1: Social exclusion is negatively correlated with mental health of youths affected by parental HIV/AIDS.*

### The mediating role of perceived stress

Social exclusion may influence perceived stress. Studies have shown that social exclusion linked to the increased stress of youths affected by parental HIV/AIDS [19–21]. Simultaneous neurophysiological evidences suggested that cognitive control-relevant brain regions (e.g., prefrontal cortex) are activated during rejection [22,23], triggering low sensory control and allowing individuals to sense more stress. Thus, social exclusion in youths affected by parental HIV/AIDS may have a positive predictive effect on perceived stress.

In addition, perceived stress has the potential to further affect the mental health of youths affected by parental HIV/AIDS. Previous studies have found a strong link between perceived stress and mental health, for example, by studying black women who had negative experiences, Catabay et al. [24] found that perceived stress significantly increased their risk of mental health symptoms such as depression and post-traumatic stress disorder. Therefore, Hypothesis 2 is further proposed:

*H2: The perceived stress of youths affected by parental HIV/AIDS has a mediating effect between social exclusion and mental health.*

### The mediating role of future orientation

Future orientation refers to an individual’s thoughts, plans, motivations, and feelings about their future [25], which are influenced by their specific living environment. Research demonstrates that generalized future-oriented worrying is typically intrusive and compulsive, often precipitated by specific stressors [26,27]. As a potent interpersonal stressor, social exclusion promotes defensive psychological responses, impairs trust, and increases future uncertainty, thereby diminishing future confidence [28]. Empirical evidence consistently shows an inverse relationship between social exclusion and future-oriented motivation --- heightened exclusion predicts lower future expectations [29].

The development of future orientation could play a crucial role in an individual’s mental health. Empirical studies have found that the positive development of future orientation is particularly important, and contributes to the psychosocial adaptation [25,30]. Zhang et al. [31] have shown that positive future orientation has a protective effect on AIDS orphans’ mental health after traumatic events. Snyder and Lopez [32] found that positive expectations always yielded higher confidence and that people who have positive expectations about the future reported more happiness and relief, more satisfaction with their quality of life, and could more easily confront adversity or difficulty in the future. Another cognitive factor related to hope and future expectation is the perceived control over the future, which was found to be positively associated with psychological well-being [8]. Thus, this research proposes Hypothesis 3:

*H3: Future orientation has a mediating effect between social exclusion and mental health of youths affected by parental HIV/AIDS.*

Lazarus and Folkman’s Stress and Coping Theory (SCT) [33] was able to provide the theoretical framework for this study, which sorts out how social support/exclusion affects an individual’s mental health. The theory states that social support is a key resource in the face of challenges and influences our stress levels. When we feel supported and able to cope, we tend to be less stressed and more hopeful about the future, which promotes individual mental health. Conversely, experiencing social exclusion can increase stress and discouragement about the future, ultimately undermining mental health. Therefore, Hypothesis 4 is proposed as follows:

*H4: Perceived stress and future orientation have a serial mediating effect between social exclusion and mental health of youths affected by parental HIV/AIDS.*

Based on the SCT, the aim of the present study is to explore the effect and mechanism of social exclusion as a negative social environmental factor on the mental health. More importantly, it attempts to explore the important intermediary factors that can mediate social exclusion and mental health of youths affected by parental HIV/AIDS.

## Method

### Participants

Participants were 331 youths from a rural county in central China, drawn from a Sino-US collaborative project 15 years prior (N = 1,600; see [34] for original recruitment procedures). The current study traced and re-contacted the original cohort; these 331 individuals constituted the successfully reached and willing participants. We excluded participants with construct-level missing (i.e., with missing data on all items within a measurement construct). Subsequently, we dealt with the missing data within a construct using a person’s mean across the available items to represent the construct, following the suggestion by Newman [35].

### Procedure

A cross-sectional follow-up survey was conducted in 2020 in Henan Province, central China, the same area of the original study. The participants in the original study all provided permission for follow-up study. The most original recruitment of participants for this study began on September 20, 2005, and the current follow-up of them began on September 28, 2020, and ended on January 19, 2021. By querying the contact information registered in orphanages and schools, we got in touch with 331 youths who completed a questionnaire through the combination of online and offline surveys. For participants who worked or went to school in other places, we sent informed and online link and invited them to participate. Before sending the survey links, we obtained participants’ permission to contact them via WeChat and Messages (the two most popular social media platforms among youths in China). They were then provided with a study description and invited to participate. For the offline survey, two trained graduate students in psychology administered face-to-face questionnaires to participants. Both online and offline surveys collected demographic information and data on social exclusion, perceived stress, future orientation, and mental health using same questionnaire and a standard instruction. The recruitment and data collection procedures were reviewed and approved by the Ethics Committee of Henan University School of Psychology (IRB 00007212; 20200315001). Written informed consent was obtained from all the participants prior to the enrollment of this study.

## Measures

### Social exclusion

A scale developed in China by Wu et al. [36] was used to measure participants’ social exclusion. The scale measured both direct exclusion and indirect exclusion (e.g., “I get unkind looks for no reason”, “People are impatient and perfunctory with my inquiries or requests.”) and demonstrated high reliability and validity in a sample of Chinese undergraduate. It consists of 19 items rated on a 5-point scale (from 1 = never to 5 = always). The total scale score ranges from 19 to 95, with higher scores indicating greater social exclusion. In the present study, the Cronbach’s alpha was 0.98.

### Perceived stress

The Scale of Perceived Stress (PSS) was used to measure perceived stress [37]. The Chinese version of the PSS (CPSS) has been validated in the literature [38].The CPSS consists of 14 items (e.g., “I felt nervous and stressed.”) rated on a 5-point scale (from 1 = never to 5 = a lot). A sum score was calculated as the scale score with higher scores indicating higher perceived stress. In the present study, the Cronbach’s alpha was 0.71.

### Future orientation

The future orientation scale (FOS) developed by Whitaker and Miller [39] was used to measure participants’ judgment and grasp of their future (e.g., “My future is what I make it”). It consists of 17 items rated on a 5-point scale (from 1 = will not happen to 5 = will definitely happen), and higher scores indicate higher control over future orientation. In the present study, the Cronbach’s alpha was 0.78.

### Mental health

The mental health scale (MHS) was used to measure mental health [40]. The MHS consists of 27 items measuring mental health status (e.g., “My life is meaningful now.”). Each item has a 5-point Likert scale (from 1 = completely untrue to 5 = completely true). A sum score was calculated as the scale score with a higher score indicating a better mental health status. The Cronbach’s alpha was 0.95 in the current study.

### Statistical analysis

Harman single-factor test was performed to detect possible common method bias [41]. The correlation and descriptive analysis of social exclusion, perceived stress, future orientation and mental health measures of the study sample was conducted. The bootstrap method was used to examine the mediation effects. In this study, common method bias test and descriptive statistical analysis were performed using SPSS24.0, and the PROCESS macro test was used to examine the mediation effect of perceived stress and future orientation.

## Results

### Preliminary analyses

As shown in Table 1, the final sample comprised 297 participants aged 22–29 years (42.76% female, 57.24% male). The sample included 129 orphans (youths who lost one or both parents due to HIV/AIDS) and 168 vulnerable youths (youths whose one or both parents were living with HIV/AIDS). Notably, all participants were confirmed HIV-negative, and the majority of affected parents contracted HIV through unsafe blood transfusion practices, a historically prevalent mode of infection in rural China during the 1990s and early 2000s. About 84.18% of the youths reported very good (61.28%) or good (22.90%) health status, and 44.44% of the youths lived in the countryside at the time of the current study.

**Table 1:** Individual characteristics of study sample.

	Overall	AIDS orphans	Vulnerable youths
***N* (%)**	297(100%)	129(43.43%)	168(56.56%)
Male	170(57.24%)	71 (55.04%)	99 (58.93%)
Female	127(42.76%)	58 (44.96%)	69 (41.07%)
**Mean age in years(SD)**	25.7(3.14)	26.66 (2.70)	25.14 (3.30)
**Self-reported health status**			
Very good	182(61.28%)	94 (72.87%)	88 (52.38%)
Good	68(22.90%)	25 (19.38%)	43 (25.60%)
Fair	37(12.46%)	7 (5.43%)	30 (17.86%)
Poor	10(3.36%)	3 (2.32%)	7 (4.16%)
**Current Working Status**			
Farming	48(16.16%)	15 (11.63%)	33 (19.64%)
College/postgraduate students	43(14.48%)	12 (9.30%)	31 (18.45%)
Odd jobs	68(22.89%)	26 (20.16%)	42 (25.0%)
Permanent jobs	111(37.37%)	61 (47.29%)	50 (29.76%)
Government employees	4(1.34%)	1 (0.78%)	3 (1.78%)
Public institution employees	23(7.74%)	14 (10.84%)	9 (5.35%)
**Current residence**			
Countryside	132(44.44%)	52 (40.31%)	80 (47.62%)
Town	50(16.84%)	24 (18.60%)	27 (16.07%)
Small-medium cities	57(19.19%)	29 (22.48%)	28 (16.67%)
Big cities	58(19.53%)	24 (18.60%)	33 (19.04%)

The common method bias test among all survey items showed that there were 13 factors with eigenvalues greater than 1, and the variation explained by the first factor was 29.16%, far less than the critical standard of 40%. The results thus indicated that common method bias was not large enough to distort the results.

Table 2 presents the means, standard deviations, and correlations for all study variables. Overall, participants reported moderate levels of social exclusion (M = 32.48, *SD* = 15.25) and perceived stress (*M* = 39.28, *SD* = 6.35), while future orientation (*M* = 57.44, *SD* = 8.15) and mental health (*M* = 93.27, *SD* = 19.08) scores indicated relatively positive outcomes. Preliminary analyses revealed no significant gender differences in any variables (*ps* > 0.05), but age showed minor correlations with other measures; thus, only age was controlled in subsequent analyses.

**Table 2:** Descriptive statistics and correlation matrix for variables.

	*M*	*SD*	1	2	3	4	5
Gender	1.43	0.50	1				
Age	25.80	3.14	−0.04	1			
Social Exclusion	32.48	15.25	−0.09	−0.16^*^	1		
Perceived Stress	39.28	6.35	0.01	−0.13^**^	0.29^***^	1	
Future Orientation	57.44	8.15	−0.07	0.14^*^	−0.38^***^	−0.57^***^	1
Mental Health	93.27	19.08	0.01	0.12^*^	−0.42^***^	−0.64^***^	0.72^***^

Note. * p < .05, ** p < .01, *** p < .001. The same as follows.

All key variables were significantly correlated (*ps* < 0.05). Social exclusion was negatively associated with future orientation (*r* = −0.38, *p* < 0.001) and mental health (*r* = −0.42, *p* < 0.001), and positively associated with perceived stress (*r* = 0.29, *p* < 0.001). Perceived stress was negatively correlated with both future orientation (*r* = −0.57, *p* < 0.001) and mental health (*r* = −0.64, *p* < 0.001). Future orientation showed a strong positive association with mental health (*r* = 0.72, *p* < 0.001).

### Serial mediation analysis

A serial mediation model was used to examine the mediation of perceived stress and future orientation in social exclusion and mental health. After controlling for age as a covariate variable, the mediation analyses were performed using the bootstrapping method with bias-corrected confidence estimates.

**Fig 1:**
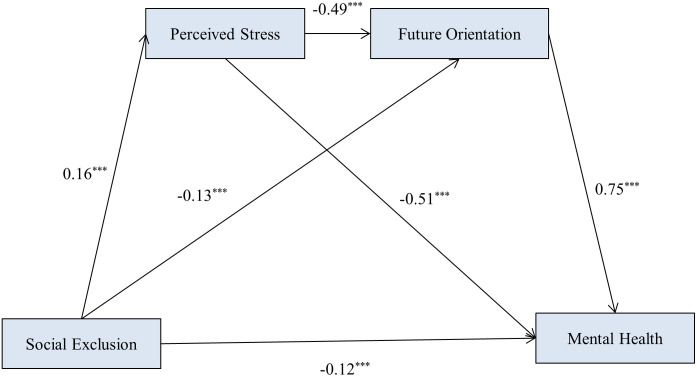
The serial mediation model.

As showed in Table 3, firstly, social exclusion has a significant impact on the mental health (*β *= −0.26, *t *= −2.92, *p* < 0.001). After the mediating variables are included, future orientation has a significant positive impact on the mental health (*β *= 0.75, *t *= 10.50, *p* < 0.001). Perceived stress not only has a significant negative impact on the mental health (*β *= −0.51, *t *= −7.35, *p* < 0.001), but also has a significant negative impact on the future orientation (*β *= −0.49, *t *= −10.17, *p* < 0.001). The social exclusion not only positively affects the perceived stress (*β *= 0.16, *t *= 4.97, *p* < 0.001), but also has a negative effect on future orientation (*β *= −0.13, *t *= −4.66, *p* < 0.001) and mental health (*β *= −0.12, *t *= −3.54, *p* < 0.001). Age as controlled variable has *p*-values greater than 0.05, indicating that it had a small effect on all four dimensions, with negligible effects in terms of the serial mediating effect (Table 4, Fig 1).

**Table 3:** Regression analysis of the serial mediation model.

Dependent	Predictors	Model Summary				
		*F*	*R* ^*2*^	*β*	SE	*t*
Mental Health	Social Exclusion	5.44	0.08	−0.26	0.09	−2.92^***^
	Age			0.04	0.02	1.91
Perceived Stress	Social Exclusion	15.94	0.09	0.16	0.03	4.97^***^
	Age			−0.01	0.01	−1.48
Future Orientation	Perceived Stress	57.72	0.37	−0.49	0.05	−10.17^***^
	Social Exclusion			−0.13	0.03	−4.66^***^
	Age			0.01	0.01	0.90
Mental Health	Perceived Stress	115.47	0.61	−0.51	0.07	−7.35^***^
	Future Orientation			0.75	0.07	10.50^***^
	Social Exclusion			−0.12	0.04	−3.54^***^
	Age			−0.01	0.01	−0.27

**Table 4:** Path coefficients of the variables in the serial mediation model.

	Route	Effect	SE	95%CI
Direct effect	Social Exclusion→Mental Health	−0.12	0.04	(−0.19, −0.05)
Indirect effect 1	Social Exclusion→Perceived Stress→Mental Health	−0.08	0.02	(−0.13, −0.05)
Indirect effect 2	Social Exclusion→Perceived Stress→Future Orientation→Mental Health	−0.06	0.02	(−0.10, −0.04)
Indirect effect 3	Social Exclusion→Future Orientation→Mental Health	−0.10	0.03	(−0.16, −0.06)

## Discussion

This study tested a serial mediation model and found that social exclusion negatively impacted mental health both directly and indirectly through increased perceived stress and reduced future orientation, with a significant chain-mediating effect of stress undermining future orientation.

The study tested several hypotheses regarding the relationships between social exclusion, perceived stress, future orientation, and mental health. First, we hypothesized that social exclusion would negatively predict mental health (H1), which was supported by the significant negative correlation. We found that exposure to social exclusion was associated with a lower level of mental health. This finding was consistent with that of Marinucci et al. [42] who demonstrated the negative relation between social exclusion and mental health among other vulnerable groups (i.e., immigrant groups). The possible explanation for the result was that social exclusion events might arouse individual negative, frustrated, and painful interpersonal experiences [43], which impaired their basic needs such as a sense of belonging, control, and self-esteem [16], thus causing negative emotions [44]. Consequently, the more social exclusion these individuals felt, the lower level their mental health would be. In sum, the social exclusion affected the individual adaptability and damaged mental health. The results of this study suggest potential strategies to support the mental health of youths affected by parental HIV/AIDS, such as addressing their basic psychological needs and reducing social exclusion. The findings have certain practical implications for promoting the mental health of these youths.

Second, we expected perceived stress to mediate the relationship between social exclusion and mental health (H2), and the mediation analysis confirmed this effect. Social exclusion was significantly and positively associated with perceived psychological stress. After encountering social exclusion, the sense of belonging decreased, an inferiority complex experienced, and it was easier to evaluate stimulus events as stress [19]. This result reveals an internal psychological pathway through which social exclusion affects mental health. It shows that perceived stress is an important intermediate factor affecting the mental health of youths affected by parental HIV/AIDS.

Finally, we proposed that future orientation would mediate the relationship between social exclusion and mental health independently (H3) and as a chain mediator (H4), and the results supported this hypothesis. Building on these findings, the results suggest that social exclusion may undermine mental health through a series of mediating mechanisms: by inducing psychological stress, eroding hope, and diminishing perceived control over one’s future. This further validates the applicability of SCT in this population. There are several possible explanations for this result. On the one hand, social exclusion experienced by these youths had a significant negative effect on their future orientation. As a negative interpersonal relationship, the social exclusion will affect individual distrust of others, cause tension and other emotions, and then aggravate the uncertainty about the future. On the other hand, individuals with negative future orientation are short of hope and expectations for the future and are more emotionally fragile. This could jeopardize their life satisfaction [45], which is one of the key indicators of psychological well-being. In addition, previous study has found that hope and positive future expectations were closely related to optimism, and were considered personality traits [46]. People with higher levels of future orientation are less sensitive to existing stress, and they might feel healthier. Study has shown that positive future orientation could be cultivated by focused training programs [47]. Data from the current study suggested that the interventions focusing on fostering optimistic future orientation would be important for youths affected by parental HIV/AIDS to promote psychologically resilient outcomes when exposed to social exclusion.

The findings of this study have a number of important implications for future mental health promotion intervention practice. First, this study showed that social exclusion, perceived stress and future orientation could, to some extent, influence the mental health of youths affected by parental HIV/AIDS. Researchers have recognized the importance of the social environment in the mental health development. To better meet the psychological development of these youths, it is first necessary to improve the social environment, including the provision of social support and services. In the socioecological system, the social environment is placed in the outermost circle [7]. Studies have found that broad social forces have more positive effects on the development of an individual with special needs [33]. Social organizations should establish social support networks and professional centers (e.g., at school, at community, at workplace) to give youths affected by parental HIV/AIDS different types of support (e.g., community health workers, peer groups, and school counselors), to maximally reduce their social exclusion during growth. Second, perceived stress and future orientation played an important mediating role between social exclusion and mental health, which means that perceived stress and future orientation is the most proximal factor that could improve mental health among youths affected by parental HIV/AIDS. It is recommended that government bodies and educational professionals focus on developing youths’ psychological assets and future cognition, with particular attention to future orientation. Practical interventions should aim to boost future confidence and motivation, facilitate realistic goal-setting, and enhance perceived control over life trajectories.

There are some limitations in the current study. First, the cross-sectional data prevent causal interpretation of the relationship between the study variables. Future studies should use a longitudinal study design to explore the causal relationship between these variables. Second, because of the unique cause of parental HIV/AIDS in the study area and the geographic location of the study sites, the sample in the current study might not be representative of youths orphaned by HIV/AIDS in China. Therefore, our ability to generalize the findings of this study to youths affected by parental HIV/AIDS in other areas is limited. Third, for those youths who were identified as vulnerable children in the original study, we did not ask the vital status of their parents because of the traumatic nature of the questions. However, based on the average survival period (from HIV diagnosis to death) of 5–10 years among people living HIV in China during the early phase of the epidemic [48], we assumed that most, if not all, of the parents who lived with HIV 15 years ago were deceased and the number of remaining vulnerable youths may be very small for any meaningful comparison between orphans and non-orphans.

## Conclusion

The present study highlights the critical need to address the mental health challenges faced by youths affected by parental HIV/AIDS in rural China, particularly those exposed to social exclusion. Our findings demonstrate that social exclusion significantly undermines mental health, with perceived stress exacerbating this negative effect, while future orientation serves as a protective psychological resource. The chain-mediating role of perceived stress and future orientation further elucidates the underlying mechanisms through which social exclusion influences mental health outcomes, aligning with the Stress and Coping Theory (SCT).

## Supporting information

S1 Appendix Ethics approvals. (PDF)

S2 Appendix Scales used. (DOCX)

S3 Appendix Raw data. (XLSX)
