# Supplementary material for: Social exclusion and mental health of youths affected by parental HIV/AIDS in China: Based on a serial mediating model
Source: PLoS One. 2025 Jul 2;20(7):e0327089. doi: 10.1371/journal.pone.0327089 (PMC12221013; doi:10.1371/journal.pone.0327089)
Supplement: S1 Appendix — (PDF) [file pone.0327089.s001.pdf]

Approval Form for Ethical Review of Psychological Research in Henan Province

Key Laboratory of Psychology and Behavior

1. Number（To be completed by the Ethics Committee）: 20200315-001

2. Research Title: Effects of social support on the development of social mentality among AIDS orphans: a 15-year follow-up study

3. Research Leader

**Name: Zhao Junfeng**	**Institution: School of Psychology, Henan University**
**Tel:+8613503780728**	**Email:** jfzhao63@hotmail.com

4. Research Program Overview

Children affected by AIDS were selected from the participants of the China-US international cooperation project 15 years ago. Through questionnaire survey, behavioral experiment, EEG experiment, this research explored the influence of different levels of social support on the mental health of AIDS orphans and their development of social mentality under different levels of social support after 15 years, and to form a coping strategy to positively improve AIDS orphans' social mentality. The recruitment of participants and data collection began in April 2020 and ended in December 2022.

5. Potential Ethical Issues and Measures to Address Them

The survey was conducted from October 2020 to February 2021 and distributed through online platforms such as WeChat and Wenjuanxing. Participants voluntarily clicked on the link to fill out the questionnaire. Before filling out the questionnaire, the purpose of the questionnaire was informed to them, and it was also made clear that clicking "submit questionnaire" was regarded as informed consent. The behavioral experiment and the electroencephalogram (EEG) experiment were carried out from March to July 2021 in a professional psychology laboratory. Participants will fill out the informed consent form before conducting the experiments. During the questionnaire survey and related experiments, participants can withdraw at any time.

6. Research Leader Assurance

I declare that the contents filled out are true and I will conduct the research in strict accordance with the relevant contents of the approval form. I also declare that I will strictly abide by the national laws and relevant regulations, while protecting the health, rights and privacy of the research subjects.

Signature: Junfeng Zhao

Date: 2020.3.15

The following content is filled in by the Ethics Committee

**7. Review Expert Opinion**<br>After review, the research value of the project is high, the research design is reasonable, the consideration of potential ethical issues is adequate, the treatment measures taken are effective and appropriate, and it meets the ethical requirements and is approved to carry out the research.<br>**Signature:** Huowang Gong<br>**Date:** 2020.3.15
**8. Ethics Committee Opinion**<br>This committee has examined the scientific value and ethical issues of the research content and agreed to the implementation of the research.<br>**Committee Chairman (signature):** yang [illegible]<br>**Ethics Committee (official seal):**

河南省心理与行为重点实验室心理学研究伦理审查审批表

1.编号（由伦理委员会填写）：20200315001	
**2.**研究名称：社会支持影响艾滋孤儿社会心态发展之十五年追踪研究	
**3.**研究负责人	
姓　名:赵俊峰	单　　位:河南大学心理学院
电　　话: **13503780728**	电子邮箱: **jfzhao63@hotmail.com**
**4.**研究方案概述<br>以 **15** 年前中美国际合作课题被试中选取受艾滋病影响儿童为被试，通过问卷调查、行为实验、脑电实验等多种技术手段和方法，探索不同社会支持水平对艾滋孤儿心理健康的影响以及十五年艾滋孤儿成年后不同社会支持水平下社会心态发展规律，形成正面提升艾滋孤儿社会心态的应对策略。本次调查招募被试和收集数据从 **2020** 年 **4** 月开始，到 **2022** 年 **12** 月结束。	
**5.**潜在的伦理议题与处理措施:<br>调查问卷从 **2020** 年 **10** 月至 **2021** 年 **2** 月进行，通过微信、问卷星等网络平台发放。参与者自愿点击链接进行问卷填写，在问卷填写前，已告知问卷目的，并告知“提交问卷”即视为知情同意。行为实验和脑电实验从 **2021** 年 **3** 月至 **7** 月进行，在专业心理学实验室中进行。参与者进行实验前将填写知情同意书。问卷调查和相关实验过程中，被试均可随时退出。	
**6.**研究负责人保证书:<br>本人声明所填写内容属实，并将严格按照审批表中有关内容从事研究。本人并表示严格遵守国家法律和有关规定，同时保护研究对象的健康、权益和隐私。<br>签　　名：赵俊峰<br>日　　期：2020 年 3 月 15 日	
以下内容由伦理委员会填写	
**7.**评审专家意见:<br>经审查，该项目的研究价值较高，研究设计合理，对潜在伦理议题的考虑充分，采取的处理措施有效得当，符合伦理要求，予以批准开展研究。<br>签　　名：[illegible]<br>日　　期：2020.3.15	
**8.**伦理委员会意见:<br>本委员会已经考察了研究内容的科研价值与伦理议题，同意该研究的实施。<br>委员会主席（签章）：[illegible]<br>伦理委员会（公章）：河南省心理与行为重点实验室伦理委员会	
