# Supplementary material for: Social exclusion and mental health of youths affected by parental HIV/AIDS in China: Based on a serial mediating model
Source: PLoS One. 2025 Jul 2;20(7):e0327089. doi: 10.1371/journal.pone.0327089 (PMC12221013; doi:10.1371/journal.pone.0327089)
Supplement: S2 Appendix — (DOCX) [file pone.0327089.s002.docx]

**Social Exclusion Questionnaire**

1. People intentionally or unintentionally avoid me when joking or playing around.
2. I become the target of malicious teasing.
3. People do not share their feelings or exchange experiences with me.
4. Others speak ill of me behind my back, affecting how people perceive me.
5. My mistakes are mocked or harshly criticized.
6. People often report my errors or oversights to authorities.
7. When I join a group conversation, the atmosphere turns cold.
8. When I might embarrass myself or make a mistake, others just wait to see me fail.
9. People mock my weaknesses, hurting me deeply.
10. I receive unfriendly stares for no apparent reason.
11. When I feel down, no one comforts or consoles me.
12. Even when I try to improve relationships, I get no positive response.
13. Even if we know each other, people don’t greet me first.
14. When I ask questions or make requests, others respond impatiently or dismissively.
15. My attempts to start conversations rarely receive warm responses.
16. People hardly notice me or know much about me.
17. Others intentionally or unintentionally keep their distance from me.
18. No matter what topic I bring up, people rarely engage in conversation.
19. My interactions with others seem shallow or short-lived.

**Mental Health Questionnaire**

1. I enjoy my current life.
2. I feel shy and uncomfortable around the opposite sex.
3. I lack motivation to study.
4. I am emotionally volatile. / I get emotional easily.
5. I have a clear direction for my post-graduation life.
6. I welcome life’s challenges.
7. My life feels meaningful right now.
8. I struggle to build healthy relationships with the opposite sex.
9. I lack self-discipline.
10. My mood is easily influenced by external factors.
11. I don’t know what I want to do after graduation.
12. I recover quickly after setbacks.
13. My college life is fulfilling.
14. I tend to avoid interpersonal issues.
15. I procrastinate on my coursework whenever possible.
16. My mood fluctuates frequently.
17. I have no long-term plans for the future.
18. Any challenge makes me feel uneasy.
19. I think college life is terrible.
20. I feel anxious around strangers.
21. I can’t focus on studying.
22. I often lose my temper and can’t control it.
23. I don’t understand the meaning of life.
24. I feel helpless when facing difficulties.
25. My life feels dull and monotonous.
26. I feel disrespected by others.
27. I lack perseverance and often leave things unfinished.

**Perceived Stress Scale (PSS)**

1. I feel upset about unexpected things happening.
2. I feel unable to control important matters in my life.
3. I feel nervous, anxious, or stressed.
4. I can successfully handle irritating issues in life. *(Reverse-scored)*
5. I feel capable of managing significant life changes effectively. *(Reverse-scored)*
6. I feel confident about handling my personal problems. *(Reverse-scored)*
7. I feel things are going my way. *(Reverse-scored)*
8. I find myself unable to complete necessary tasks.
9. I can resolve unpleasant things in life. *(Reverse-scored)*
10. I feel in control of my life. *(Reverse-scored)*
11. I feel angry when things happen beyond my control.
12. I keep thinking about unfinished tasks I must complete.
13. I feel in control of how I spend my time. *(Reverse-scored)*
14. I feel problems are piling up without solutions.

**Future Orientation Questionnaire**

**Part 1: Open-Ended Question**

1. What do you want to be when you grow up? _____________________

**Part 2: Confidence in Future Outcomes**

*(How sure are you that...)*
2. You will be able to handle problems you encounter in the future?
3. You will manage schoolwork/work responsibilities as you grow older?
4. You will always have friends and people who care about you?
5. You will avoid serious troubles in the future?
6. Your life will be happy in the future?
7. You will be able to do things that interest you?

**Part 3: Likelihood of Future Events**

*(How likely is it that...)*
8. You will pursue higher education?
9. You will find a good job?
10. You will own a car?
11. You will live in a desirable place?

**Part 4: Beliefs About the Future**

*(Rate your agreement with:)*
12. My future is largely in my own hands.
13. I can do whatever I set my mind to.
14. I create my own future.
15. I feel confident about my future.
16. Sometimes I feel there’s nothing to look forward to. *(Reverse-scored)*
17. I take life one day at a time.
18. Worrying about the future is pointless—what will be will be. *(Reverse-scored)*
